# Conservative and Minimally Invasive Interventions for Temporomandibular Disorders: Protocol for a Systematic Review of Randomized Controlled Trials

**DOI:** 10.3390/medsci14010108

**Published:** 2026-02-23

**Authors:** Eugenia Larisa Tarevici, Oana Tanculescu, Alina Mihaela Apostu, Alice-Teodora Rotaru-Costin, Sorina Mihaela Solomon, Adrian Doloca, Marina Cristina Iuliana Iordache

**Affiliations:** Grigore T. Popa University of Medicine and Pharmacy Iasi, 16 Universitatii Street, 700115 Iasi, Romania; eugenia-tarevici@umfiasi.ro (E.L.T.); alice-teodora.rotaru@umfiasi.ro (A.-T.R.-C.); sorina.solomon@umfiasi.ro (S.M.S.); adrian.doloca@umfiasi.ro (A.D.); marina.iordache@umfiasi.ro (M.C.I.I.)

**Keywords:** temporomandibular disorders, systematic review protocol, randomized controlled trials, conservative treatment, minimally invasive therapy

## Abstract

**Background:** Temporomandibular disorders (TMDs) are common musculoskeletal conditions associated with pain, functional limitation, and reduced quality of life (QoL). Despite the widespread use of conservative and minimally invasive treatments, the available evidence remains fragmented across heterogeneous interventions, diagnostic criteria, and outcome measures, limiting comparative interpretation and clinical applicability. **Objectives:** The primary objective of this systematic review is to evaluate the effectiveness of conservative and minimally invasive interventions for pain reduction in adult patients with temporomandibular disorders. Secondary objectives include assessing effects on mandibular function and QoL and exploring differences across intervention categories, TMD subtypes, diagnostic criteria, and follow-up durations. **Methods:** This protocol is registered in the International Prospective Register of Systematic Reviews (PROSPERO; CRD420251250251) and adheres to the Preferred Reporting Items for Systematic Review and Meta-Analysis Protocols (PRISMA-P) guidelines. A systematic search will be conducted in PubMed/MEDLINE, Web of Science, Scopus, Embase, and Cochrane Central Register of Controlled Trials (CENTRAL) for randomized controlled trials (RCTs) published from 1 January 2015, up to the date of study initiation, using controlled vocabulary terms and free-text keywords combined with Boolean operators. Eligible studies will include adult patients (≥18 years) diagnosed with temporomandibular disorders using validated diagnostic criteria and treated with conservative or minimally invasive interventions, compared with placebo/sham, no treatment or usual care, or active comparators, in accordance with the PICOS framework. Two reviewers will independently screen studies and extract data, with disagreements resolved by consensus or consultation with a third reviewer; the study selection process will be documented using a PRISMA 2020 flow diagram. Interventions will be synthesized within predefined clusters (e.g., physical and manual therapies, occlusal splint therapy, physical agent modalities, and minimally invasive joint procedures). Risk of bias will be assessed using the revised Cochrane Risk of Bias tool (RoB 2). The primary outcome will be pain intensity, while secondary outcomes will include mandibular function and QoL. Where appropriate, meta-analysis using a random-effects model will be performed; otherwise, a structured narrative synthesis will be provided. **Expected Impact:** The systematic review is expected to deliver an updated and methodologically rigorous synthesis of evidence on conservative and minimally invasive interventions for TMDs. By addressing existing research gaps such as the fragmentation of evidence across intervention types, heterogeneity in diagnostic criteria, and variability in outcome measures, this review will support evidence-based clinical decision-making and identify priorities for future research.

## 1. Introduction

Temporomandibular disorders (TMDs) comprise a heterogeneous group of musculoskeletal and neuromuscular conditions affecting the temporomandibular joint (TMJ), masticatory muscles, and associated craniofacial structures [1,2]. TMDs are characterised by a wide spectrum of symptoms, the most relevant of which are pain in the jaw and preauricular regions, limited or asymmetric mandibular movements, joint sounds, muscle tenderness, and functional impairment. Headache, neck pain, sleep disturbances, and psychosocial distress accompany these symptoms, playing an important role in altering health-related quality of life (QoL) [3,4]. Due to their multifactorial etiology and variable clinical presentation, TMDs represent a frequent and challenging condition in daily dental and medical practice [5,6,7].

The conceptual understanding and knowledge of TMD has shifted over the past decades, from a mechanistic paradigm focused on occlusal and morphological factors to a biopsychosocial model. This paradigm suggests that TMD etiology is multifactorial, resulting from an interconnected relationship among biological factors, mechanical factors, and psychosocial factors [1,2]. Consequently, TMD is no longer viewed as a monolithic disease entity but as a cluster of overlapping conditions, mainly divided into intra-articular and muscular disorders, each calling for personalized management strategies [1,5].

Reflecting this paradigm shift, current clinical guidelines and consensus statements consistently advocate for conservative, reversible, and patient-oriented interventions as the first-line approach for TMD management [8,9]. Conservative management aims to relieve pain, restore function, and address contributing behavioral and psychosocial factors while reducing the risk of overtreatment. Common conservative interventions include patient education, behavioral modification, physical therapy, pharmacotherapy, and occlusal splint therapy [9,10,11,12,13,14,15]. Physical agent modalities such as photobiomodulation and transcutaneous electrical nerve stimulation are increasingly used for their analgesic and anti-inflammatory effects [16,17,18,19].

Despite the effectiveness of conservative therapies in many patients, a subset of individuals experience persistent or refractory symptoms. In such cases, minimally invasive interventions play an important role as a bridge between conservative management and open joint surgery [20]. Techniques such as arthrocentesis and intra-articular injections have become widely accepted because of their potential to modulate joint inflammation, wash out inflammatory mediators, improve lubrication, influence neuromuscular function, and promote tissue repair with a favorable risk–benefit profile [21,22,23]. Furthermore, over the last decade, advances in regenerative medicine and rehabilitation technologies have continued to enlarge the therapeutic arsenal for TMDs. Randomized controlled trials have investigated a wide array of minimally invasive and technology-assisted interventions, including arthrocentesis, intra-articular injections, platelet-rich plasma, hyaluronic acid, botulinum toxin administration, and dry needling [24,25,26]. These interventions target distinct biological and neuromuscular mechanisms and are often combined with conventional conservative therapies, indicating an increasing interest in multimodal, customized therapeutic strategies [27,28,29].

This diversity of conservative and minimally invasive therapeutic modalities reflects real-world clinical practice, in which treatment strategies are frequently individualized and multimodal rather than based on a single isolated intervention [30]. While this complexity accurately mirrors contemporary TMD management, it has also contributed to a fragmented body of evidence, characterized by substantial heterogeneity in diagnostic criteria (e.g., RDC/TMD versus DC/TMD), intervention protocols, and outcome measures [31,32,33,34,35]. Consequently, many existing systematic reviews focus on isolated interventions or specific TMD subtypes, limiting the ability to compare the relative effectiveness of the broad spectrum of conservative and minimally invasive strategies within an integrated clinical framework [28,36,37].

Despite the growing number of randomized controlled trials, the available evidence remains challenging to translate into coherent clinical decision-making pathways. In particular, the lack of integrated syntheses that compare multiple conservative and minimally invasive strategies within a unified analytical framework is a critical gap in the current literature. This challenge is further amplified by heterogeneity in diagnostic frameworks, including the transition from the Research Diagnostic Criteria for TMD (RDC/TMD) to the Diagnostic Criteria for TMD (DC/TMD), as well as inconsistencies in outcome selection, follow-up duration, and reporting across studies [2].

In this context, there is a clear need for a comprehensive and methodologically rigorous synthesis of randomized controlled trials evaluating conservative and minimally invasive interventions for TMDs. Addressing such a broad clinical scope requires a predefined analytical framework capable of managing clinical and methodological heterogeneity while prioritizing clinically meaningful outcomes. Publishing the present protocol before data synthesis aims to enhance transparency, reduce the risk of selective reporting, and allow critical appraisal of the review methodology before undertaking the substantial analytical work required for a full systematic review and meta-analysis.

In addition, this synthesis can guide practitioners in selecting the most effective therapeutic options and identifying which patient subgroups may benefit most from particular interventions, thereby facilitating personalized, evidence-based care and highlighting critical areas for future research.

**Aim and Objectives:** The primary objective of the planned systematic review is to evaluate the effectiveness of conservative and minimally invasive interventions for pain reduction in adult patients with temporomandibular disorders.

Secondary objectives include assessing effects on mandibular function and QoL and exploring differences across intervention categories, diagnostic criteria, and follow-up durations.

## 2. Materials and Methods

### 2.1. Protocol Registration and Reporting Standards

This study is designed as a systematic review of randomized controlled trials (RCTs) and follows a prospectively registered protocol. The protocol was registered a priori in the International Prospective Register of Systematic Reviews (PROSPERO; registration number CRD420251250251) [38] and was developed in accordance with the Preferred Reporting Items for Systematic Review and Meta-Analysis Protocols (PRISMA-P) guidelines [39]. The review will be conducted and reported in line with the PRISMA 2020 statement. Any protocol amendments will be documented and updated in the PROSPERO register.

### 2.2. Eligibility Criteria (PICOS Framework)

Eligibility criteria were defined a priori according to the PICOS framework (Population, Intervention, Comparator, Outcomes, and Study design).

#### 2.2.1. Population

Studies including adult participants (≥18 years) diagnosed with temporomandibular disorders (TMD) will be considered eligible. Eligible diagnoses will encompass muscular (myogenous), intra-articular (arthrogenous), or mixed TMDs, based on validated diagnostic criteria. Both the Research Diagnostic Criteria for TMD (RDC/TMD) and the Diagnostic Criteria for TMD (DC/TMD) will be accepted, acknowledging their use across different time periods and clinical contexts.

Studies including mixed populations (e.g., concomitant headache or cervical pain) will be eligible only if data specific to TMD-related outcomes can be clearly extracted. Studies focusing exclusively on pediatric or adolescent populations (<18 years) will be excluded. Studies involving patients with TMD secondary to systemic inflammatory diseases (e.g., rheumatoid arthritis), tumors, fractures, or recent trauma or surgery will also be excluded.

#### 2.2.2. Interventions

Conservative and minimally invasive interventions for the management of temporomandibular disorders will be eligible for inclusion.

Conservative interventions will include non-invasive, reversible therapeutic approaches commonly recommended as first-line management for TMDs, including, but not limited to:

- physical therapy (active or passive);
- manual therapy techniques;
- occlusal splint therapy (e.g., stabilization splints, repositioning splints, anterior bite planes);
- laser therapy, including low-level laser therapy modalities (photobiomodulation) and high-intensity laser modalities;
- transcutaneous electrical nerve stimulation (TENS);
- acupuncture and related physical modalities;
- psychosocial or cognitive-behavioral interventions (e.g., biofeedback) when combined with physical therapies;
- multimodal conservative treatment programs combining two or more of the above interventions.

Minimally invasive interventions will be defined as procedures involving limited tissue penetration and a favorable risk–benefit profile, positioned between conservative care and open joint surgery. These may include, but are not limited to:

- temporomandibular joint (TMJ) arthrocentesis (with or without lavage);
- intra-articular injections, including: hyaluronic acid (HA), platelet-rich plasma (PRP) or other autologous blood-derived products (e.g., PRGF), and corticosteroids;
- injectable neuromodulatory therapies, specifically intramuscular or periarticular botulinum toxin injections, when used for the management of TMD-related pain or dysfunction;
- dry needling and other technology-assisted interventions investigated in randomized controlled trials.

Studies evaluating combined conservative and minimally invasive interventions will also be eligible for inclusion. Interventions involving open TMJ surgery (e.g., discectomy, arthroplasty) or exclusive pharmacological management (systemic drugs) without concurrent physical therapy will be excluded.

For analytical purposes, individual interventions will be grouped a priori into predefined clusters based on their primary therapeutic mechanism, including: (i) physical and manual therapies, (ii) occlusal splint therapies, (iii) physical agent modalities (e.g., laser therapy, TENS), (iv) conservative traditional modalities such as acupuncture, (v) minimally invasive neuromuscular interventions (e.g., dry needling, botulinum toxin), and (vi) minimally invasive joint procedures (e.g., arthrocentesis and intra-articular injections).

#### 2.2.3. Comparators

Eligible comparators will include but will not be limited to placebo or sham interventions (e.g., sham laser, saline injection), no treatment or usual care (e.g., patient education/counseling), and active comparators, including alternative conservative or minimally invasive treatment. Studies comparing different active interventions within the same therapeutic category or across different categories will also be eligible.

#### 2.2.4. Outcomes

Studies will be eligible if they report at least one of the primary or secondary clinical outcomes of interest.

The primary outcome of interest will be pain intensity, assessed using validated instruments such as the Visual Analog Scale (VAS) or Numeric Rating Scale (NRS).

Secondary outcomes will include mandibular function and patient-reported QoL. The secondary outcomes will include:

- functional jaw outcomes—including measures of jaw mobility (e.g., maximum mouth opening [MMO] in mm), lateral/protrusive movements, and functional limitation indices (e.g., Jaw Functional Limitation Scale [JFLS]);
- patient-reported global improvement or treatment success—preferably assessed using validated instruments, such as the Patient Global Impression of Change (PGIC);
- quality of life (QoL)—related to orofacial or general health, assessed using validated instruments (e.g., OHIP-14, SF-36, EQ-5D);
- adverse events—such as treatment-related complications or side effects;
- psychosocial outcomes—such as anxiety, depression, or pain-related distress (e.g., PHQ-9, GAD-7);
- sleep quality—assessed using validated tools (e.g., PSQI);
- muscle tenderness—assessed using palpation sensitivity, measured clinically or by algometry (Pressure Pain Threshold).

When multiple instruments are used to assess patient-reported global improvement, validated scales (e.g., PGIC) will be prioritized for quantitative synthesis, while non-validated or study-specific measures will be summarized narratively.

Studies reporting outcomes at different follow-up durations will be eligible, and outcome measures will be categorized by assessment timing.

Not all secondary outcomes are expected to be amenable to quantitative synthesis; where appropriate, these outcomes will be summarized narratively.

#### 2.2.5. Study Design

Only randomized controlled trials (RCTs) will be eligible for inclusion, with both parallel-group and crossover randomized designs being accepted. Quasi-randomized trials, non-randomized studies, observational designs, case series, case reports, narrative reviews, systematic reviews, and conference abstracts will be excluded to ensure a high level of evidence.

#### 2.2.6. Language and Publication Period

No language restrictions will be applied during the literature search. Screening will be conducted based on titles and abstracts available in English. When necessary, AI-assisted translation tools may be used to support full-text assessment and data extraction for studies published in other languages.

The review will include studies published from 1 January 2015 up to the date of study initiation, in order to focus on contemporary evidence reflecting current therapeutic strategies in temporomandibular disorders, while acknowledging the transition following the introduction of the Diagnostic Criteria for Temporomandibular Disorders (DC/TMD) and the continued use of both DC/TMD and RDC/TMD in clinical research during this period.

### 2.3. Information Sources

A comprehensive literature search will be conducted in the following electronic databases: PubMed/MEDLINE, Embase, Web of Science Core Collection, Scopus, and the Cochrane Central Register of Controlled Trials (CENTRAL). In addition, the reference lists of all included studies and relevant systematic reviews will be manually screened (“snowballing”) to identify additional eligible studies not captured by the electronic search. The review will primarily focus on randomized controlled trials published in peer-reviewed journals. Searches of the grey literature will therefore be limited. However, clinical trial registries (e.g., ClinicalTrials.gov) will be screened to identify completed trials with available results that have not yet been published.

### 2.4. Search Strategy

The search strategy will be developed in collaboration with content and methodology experts to ensure sensitivity and relevance. Database-specific search strategies will combine controlled vocabulary terms (e.g., Medical Subject Headings [MeSH] in PubMed and Emtree terms in Embase) with free-text keywords using Boolean operators (AND, OR).

The core search concepts will include, but will not be limited to:

- Population: “Temporomandibular Joint Disorders”, “TMD”, “TMJ dysfunction”, “Craniomandibular disorders”, “Myofascial pain”.
- Interventions: “Conservative treatment”, “Physical therapy”, “Occlusal splints”, “Low-level light therapy”, “Acupuncture”, “Arthrocentesis”, “Injections, Intra-Articular”, “Hyaluronic Acid”, “Platelet-rich plasma”.
- Study design: “Randomized Controlled Trial”, “Clinical Trial”, “Random Allocation”.

Search strategies will be adapted for each database’s syntax to account for differences in indexing and search syntax. No restrictions on study outcomes will be applied during the search phase to maximize sensitivity. The full search strategy for at least one electronic database will be provided as Supplementary Material.

### 2.5. Study Selection

All records identified through the electronic searches will be imported into a reference management software, and duplicates will be removed prior to screening.

Study selection will be conducted in two sequential stages:

- In the first stage, titles and abstracts will be independently screened by two reviewers to assess potential eligibility according to the predefined inclusion and exclusion criteria.
- In the second stage, the full texts of potentially eligible studies will be retrieved and independently assessed for final inclusion.

Any disagreements between the reviewers at either stage will be resolved through discussion and consensus. If consensus cannot be reached, a third reviewer will be consulted. The study selection process will be documented using a PRISMA 2020 flow diagram, detailing the number of records identified, screened, excluded, and included at each stage, together with reasons for exclusion at the full-text level.

### 2.6. Data Extraction and Preparation for Data Synthesis

Data extraction will be performed independently by two reviewers using a standardized, pilot-tested data extraction form. The extraction form will be developed a priori and refined through calibration exercises to ensure consistency and completeness.

The following data items will be extracted from each included study, where available:

- study characteristics—authors, year of publication, country, study design, funding source;
- participant characteristics—sample size, age, sex distribution, diagnostic criteria used (RDC/TMD or DC/TMD), TMD subtype, and duration of symptoms;
- intervention characteristics—type of conservative or minimally invasive intervention, treatment protocol, dosage or frequency, duration, and technical parameters;
- comparator characteristics—description of the control or comparison intervention;
- outcomes measures, including baseline and follow-up data for pain intensity (VAS/NRS), mandibular function (e.g., MMO), and QoL, together with reported adverse events;
- follow-up duration and attrition rates.

If required data are missing or incompletely reported (e.g., standard deviations), attempts will be made to estimate these values from other available statistics (e.g., *p*-values, confidence intervals) in accordance with established methodological recommendations. Extracted data will be organized to facilitate qualitative synthesis and, where appropriate, quantitative analysis.

### 2.7. Data Synthesis and Statistical Analysis

A qualitative and, where appropriate, quantitative synthesis of the included studies will be performed. Quantitative analyses will be conducted primarily using Review Manager (RevMan; The Cochrane Collaboration), including the desktop (v5.4) or web-based implementation, or equivalent meta-analysis software.

#### 2.7.1. Quantitative Synthesis

When studies are sufficiently homogeneous in terms of population characteristics, interventions, comparators, and outcome measures, a meta-analysis will be conducted, including head-to-head comparisons of therapeutic interventions. Pooled effect estimates will be calculated using a random-effects model, given the anticipated clinical and methodological heterogeneity among studies.

For continuous outcomes (e.g., pain intensity, MMO), effect sizes will be reported as mean differences (MDs) or standardized mean differences (SMDs), with corresponding 95% confidence intervals (CIs). For dichotomous outcomes (e.g., treatment success vs. failure, presence of adverse events), risk ratios (RRs) or odds ratios (ORs) with 95% CIs will be calculated.

Statistical heterogeneity will be assessed using the Chi-squared test and quantified using the I^2^ statistic. In the presence of substantial heterogeneity, potential sources will be explored through subgroup analyses or, where appropriate, results will be synthesized narratively. Thresholds for interpretation will follow Cochrane guidance: I^2^ values of 0% to 40% are non-important, 30% to 60% represent moderate heterogeneity, 50% to 90% substantial heterogeneity, and 75% to 100% considerable heterogeneity, with values greater than 50% reflecting notable heterogeneity.

Publication bias will be assessed by visual inspection of funnel plots and Egger’s test, where appropriate, by statistical tests, provided that a sufficient number of studies are available for quantitative synthesis.

#### 2.7.2. Narrative Synthesis

If meta-analysis is not possible due to substantial heterogeneity or insufficient data, a structured narrative synthesis will be conducted. Studies will be grouped according to intervention category (e.g., conservative therapies, minimally invasive interventions), comparator type, and outcome domain. The narrative synthesis will follow established methodological guidance such as the Synthesis Without Meta-analysis (SWiM) reporting framework, with emphasis on patterns of effects, consistency across studies, and clinical relevance.

#### 2.7.3. Subgroup and Sensitivity Analyses

Where data permit, subgroup analyses will be performed based on:

- type of TMD (myogenous, arthrogenous, mixed);
- category of intervention (conservative vs. minimally invasive);
- diagnostic criteria used (DC/TMD vs. RDC/TMD);
- duration of follow-up (short-term ≤ 3 months, mid-term 3–6 months, long-term ≥ 6 months).

Sensitivity analyses may be conducted to explore the impact of studies at high risk of bias on pooled estimates and to assess the robustness of the findings by excluding small-sample studies or studies with imputed data.

### 2.8. Risk of Bias Assessment

The risk of bias of the included RCTs will be independently assessed by two reviewers using the revised Cochrane Risk of Bias tool for randomized trials (RoB 2) [40]. The tool evaluates potential sources of bias across five domains:

- bias arising from the randomization process;
- bias due to deviations from intended interventions;
- bias due to missing outcome data;
- bias in measurement of the outcome;
- bias in the selection of the reported result.

Each domain will be judged as “low risk of bias,” “some concerns,” or “high risk of bias” based on the RoB 2 signaling questions and algorithmic guidance. In accordance with the RoB 2 framework, an overall risk-of-bias judgment will be assigned for each outcome within each study.

Disagreements between reviewers will be resolved through discussion and consensus. If consensus cannot be reached, a third reviewer will be consulted. The results of the risk of bias assessment will be summarized using both tabular and graphical formats and will be considered in the interpretation of findings, including subgroups and sensitivity analyses where applicable.

### 2.9. Certainty of Evidence

The certainty of the evidence for each primary and secondary outcome will be independently assessed by two reviewers using the Grading of Recommendations Assessment, Development and Evaluation (GRADE) approach [41]. The GRADE methodology evaluates the overall certainty of evidence based on the following domains:

- risk of bias;
- inconsistency of results;
- indirectness of evidence;
- imprecision;
- publication bias.

For each outcome, the certainty of evidence will be rated as high, moderate, low, or very low. Evidence derived from randomized controlled trials will initially be considered high certainty and may be downgraded if serious limitations are identified in any GRADE domains. Assessments of risk of bias will be informed by the results of the RoB 2 evaluation.

The GRADE approach will be applied to outcomes synthesized quantitatively and, where appropriate, to outcomes summarized through structured narrative synthesis. Summary of Findings (SoF) tables will be generated using GRADEpro GDT software to present key outcomes, pooled effect estimates (where applicable), and corresponding GRADE ratings in a transparent and clinically interpretable format.

### 2.10. Ethics and Dissemination

Ethical approval is not required for this systematic review, as it will involve the synthesis of data from previously published and ethically approved studies. The results of the review will be disseminated through publication in a peer-reviewed, open-access journal and through presentations at national and international scientific conferences. Any amendments to the protocol will be documented and updated in the PROSPERO registry to ensure transparency.

## 3. Strengths and Limitations of the Protocol

This protocol has several strengths. First, it is prospectively registered in PROSPERO, which enhances transparency and reduces the risk of selective reporting and post hoc methodological modifications. Second, the review will be conducted in accordance with PRISMA-P guidelines and will be restricted to randomized controlled trials, thereby prioritizing the highest level of evidence available for therapeutic interventions. Third, the inclusion of both conservative and minimally invasive treatment approaches reflects contemporary clinical practice and allows a comprehensive synthesis across therapeutic categories commonly used within multimodal management strategies for temporomandibular disorders. The planned use of the revised Cochrane Risk of Bias tool (RoB 2) and the GRADE approach further strengthens the methodological rigor of the review, supporting balanced interpretation of findings based on the quality and certainty of the underlying evidence.

Several limitations should also be acknowledged. Significant clinical and methodological heterogeneity is anticipated among the included studies, particularly with respect to intervention protocols, outcome measures, and follow-up durations. When such heterogeneity precludes quantitative synthesis, results will be summarized using a structured narrative approach in accordance with established methodological guidance (SWiM). Although no language restrictions will be applied during the screening phase, the inclusion of full-text studies may be influenced by practical constraints related to data extraction and synthesis. Any potential impact of language-related limitations will be transparently reported and considered when interpreting the findings. Finally, restricting the search to studies published from 2015 onward may exclude earlier foundational research; however, this timeframe was intentionally selected to focus on contemporary evidence generated following the introduction of the Diagnostic Criteria for Temporomandibular Disorders (DC/TMD), thereby improving diagnostic consistency and clinical relevance.

## 4. Discussion

Despite the increasing number of randomized controlled trials evaluating conservative and minimally invasive interventions for temporomandibular disorders, the interpretation of available evidence remains challenging. One of the principal issues anticipated in the present review is the substantial clinical and methodological heterogeneity across trials, particularly regarding diagnostic criteria (the transition from RDC/TMD to DC/TMD), intervention protocols (e.g., laser parameters, splint designs, injection substances), outcome definitions, and follow-up durations. Such variability may limit the feasibility of quantitative synthesis (meta-analysis) for certain comparisons and necessitate the use of structured narrative synthesis (SWiM) to ensure meaningful clinically appropriate interpretation of findings.

Variability in reported treatment effects is likely to reflect not only differences in intervention-specific mechanisms but also patient-related factors, including TMD subtype (myogenous vs. arthrogenous), symptom chronicity, psychosocial burden, and the presence of overlapping pain conditions. Conservative interventions, in particular, may involve both specific therapeutic effects and non-specific contextual influences, such as patient expectation and therapeutic alliance, which are not uniformly controlled across randomized designs. Similarly, the effectiveness of minimally invasive procedures may depend on disease stage and biological characteristics of injectable agents (e.g., hyaluronic acid molecular weight, leukocyte content in PRP), highlighting the importance of careful clinical stratification when interpreting pooled or comparative outcomes.

Another anticipated challenge concerns the potential dissociation between improvements in pain intensity and functional outcomes. Interventions may yield significant reductions in subjective pain measures (VAS/NRS) while demonstrating more modest changes in objective functional parameters, such as MMO. This observation underscores the multidimensional nature of temporomandibular disorders and supports the inclusion of QoL measures as key outcomes to capture patient-centered benefits.

By integrating rigorous risk-of-bias assessment (RoB 2) with the certainty-of-evidence grading (GRADE), this review aims to address methodological limitations that may have influenced prior syntheses. Planned subgroup and sensitivity analyses may further help identify patient profiles and intervention categories associated with more consistent therapeutic responses, thereby supporting a more personalized and evidence-based approach to TMD management.

## 5. Conclusions

This protocol outlines a rigorous and transparent methodological framework for a systematic review of randomized controlled trials evaluating conservative and minimally invasive interventions for temporomandibular disorders. By synthesizing contemporary evidence across a broad range of non-surgical therapeutic approaches, the planned review aims to address a relevant gap in the current literature regarding comparative effectiveness and clinical applicability. The findings are expected to support evidence-based clinical decision-making, inform guideline development, and highlight priority areas for future research within the multidisciplinary management of temporomandibular disorders.

## Data Availability

The original contributions presented in this study are included in the article/supplementary material. Further inquiries can be directed to the corresponding author(s).

## Supplementary Materials

The following supporting information can be downloaded at https://www.mdpi.com/article/10.3390/medsci14010108/s1. Supplementary Material S1: PubMed Search Stratege.

## Abbreviations

The following abbreviations are used in this manuscript:

TMDs	Temporomandibular disorders
TMJ	Temporomandibular joint
RCTs	Randomized controlled trials
PRISMA-P	Preferred Reporting Items for Systematic Review and Meta-Analysis Protocols
RoB 2	Cochrane Risk of Bias tool
NSAIDs	Nonsteroidal Anti-Inflammatory Drugs
TENS	Transcutaneous electrical nerve stimulation
RDC/TMD	Research Diagnostic Criteria for Temporomandibular Disorders
DC/TMD	Diagnostic Criteria for Temporomandibular Disorders
HA	Hyaluronic acid
PRP	Platelet-rich plasma
PRGF	Plasma Rich in Growth Factors
VAS	Visual Analog Scale
NRS	Numeric Rating Scale
MMO	Maximum mouth opening
JFLS	Jaw Functional Limitation Scale
QoL	Quality of life
OHIP-14	Oral Health Impact Profile-14
SF-36	36-Item Short Form Health Survey
EQ-5D	Health Questionnaire EQ-5D—EuroQol Group
PHQ-9	Patient Health Questionnaire-9
GAD-7	General Anxiety Disorder-7
PSQI	Pittsburgh Sleep Quality Index
MD	Mean differences
SMD	Standardized mean differences
CI	Confidence interval
RR	Risk ratio
OR	Odds ratio
SWiM	Synthesis Without Meta-analysis
GRADE	Grading of Recommendations Assessment, Development and Evaluation
SoF	Summary of Findings
