# Supplementary material for: Conservative and Minimally Invasive Interventions for Temporomandibular Disorders: Protocol for a Systematic Review of Randomized Controlled Trials
Source: Med Sci (Basel). 2026 Feb 23;14(1):108. doi: 10.3390/medsci14010108 (PMC13027864; doi:10.3390/medsci14010108)
Supplement: Supplementary file 1 [file medsci-14-00108-s001.zip › medsci-4102162-supplementary.pdf]

Supplementary Material S1

PubMed Search Strategy

The PubMed search strategy was developed to identify randomized controlled trials evaluating conservative and minimally invasive interventions for temporomandibular disorders.

The strategy combines Medical Subject Headings (MeSH) and free-text terms related to the population, interventions, and study design, using Boolean operators (AND, OR). No outcome-based restrictions were applied at the search stage to maximize sensitivity.

Search string used in PubMed/MEDLINE

("Temporomandibular Joint Disorders"[Mesh] OR "Temporomandibular Disorders"[tiab] OR TMD[tiab] OR "TMJ dysfunction"[tiab] OR "Craniomandibular disorders"[tiab] OR "Myofascial pain"[tiab]) AND ("Physical Therapy Modalities"[Mesh] OR "Physical therapy"[tiab] OR "Physiotherapy"[tiab] OR "Manual therapy"[tiab] OR "Occlusal Splints"[Mesh] OR "Occlusal splint"[tiab] OR "Laser Therapy"[Mesh] OR photobiomodulation[tiab] OR "Low level laser therapy"[tiab] OR "Transcutaneous Electric Nerve Stimulation"[Mesh] OR TENS[tiab] OR "Acupuncture Therapy"[Mesh] OR acupuncture[tiab] OR "Dry needling"[tiab] OR "Arthrocentesis"[Mesh] OR "Injections, Intra-Articular"[Mesh] OR "Hyaluronic Acid"[Mesh] OR "Platelet-Rich Plasma"[Mesh] OR PRP[tiab] OR "Botulinum Toxins"[Mesh]) AND ("Randomized Controlled Trial"[Publication Type] OR randomized[tiab])

Limits:

Publication date: January 1, 2015 to date of study initiation

Species: Humans

Age: Adults (≥18 years)

Notes: The search strategy will be adapted for other databases (Embase, Web of Science, Scopus, CENTRAL) using database-specific controlled vocabulary (e.g., Emtree) and syntax. Reference lists of included studies and relevant systematic reviews will be manually screened to identify additional eligible trials.
